# The signal peptide of staphylococcal protein A alters the multimeric states of PepV

**DOI:** 10.1128/spectrum.01778-25

**Published:** 2025-10-27

**Authors:** Muhammad S. Azam, Dominique Missiakas

**Affiliations:** 1 Howard Taylor Ricketts Laboratory, Department of Microbiology, The University of Chicago 2462, Chicago, Illinois, USA; Cinvestav-IPN, Mexico City, Mexico

**Keywords:** *Staphylococcus aureus*, YSIRK secretion motif, PepV, staphylococcal protein A

## Abstract

Signal peptides (SPs) have traditionally been recognized as molecular address tags that direct protein translocation between subcellular compartments. However, emerging evidence suggests that they serve additional functions beyond their canonical role in protein trafficking. In this study, we investigate the signal peptide of staphylococcal protein A (SpA), which contains the YSIRK/GXXS motif and facilitates translocation at bacterial cross-walls. Our findings reveal a previously unrecognized regulatory function of the SpA SP: it modulates the multimerization and enzymatic activity of PepV, a SecA-associated peptidase implicated in surface protein display. While PepV primarily exists as a dimer, it can also form higher-order assemblies, including tetramers, though the functional significance of these multimeric forms remains unclear. We demonstrate that the SpA SP inhibits the formation of PepV tetramers and higher-order oligomers while also suppressing its dipeptidase activity. In contrast, the canonical Sec signal peptide from SasF reduces PepV enzymatic activity without affecting its oligomerization. These findings highlight a novel regulatory dimension of signal peptides, illustrating their ability to modulate enzyme function and protein oligomerization. This expands their functional repertoire beyond protein trafficking, revealing a previously unappreciated layer of cellular regulation.

**IMPORTANCE:**

Signal sequences have traditionally been regarded as address tags that direct protein precursors to their appropriate cellular or extracellular destinations. Emerging evidence indicates that these sequences not only influence targeting and membrane insertion but also can retain functional roles after cleavage from the parent protein. Here, we present evidence for a previously unrecognized regulatory function of an uncommon Gram-positive signal peptide, demonstrating its ability to modulate both protein multimerization and enzymatic activity. These findings suggest that some signal peptides may serve broader regulatory roles beyond their conventional function in protein transport.

## INTRODUCTION

The signal hypothesis, introduced by Blobel and Sabatini in their seminal work, revolutionized our understanding of protein targeting within the secretory pathway (1). Signal peptides (SPs), functioning as address tags, play critical roles in directing proteins to specific membrane destinations and facilitating their insertion. Typically, signal sequences share a conserved tripartite structure: a positively charged N-terminus, a central hydrophobic region, and a polar segment containing the cleavage site for signal peptidase (2, 3).

In the gram-positive pathogen *Staphylococcus aureus*, signal sequences exhibit considerable diversity, depending on the pathway utilized, whether the canonical Sec pathway, the Tat pathway, or the accessory Sec pathway (4). Even within the canonical Sec pathway, notable differences in signal sequences are evident. For instance, surface proteins often contain extended signal peptides featuring the YSIRK/GxxS motif, which targets precursor proteins to the cross-wall of dividing cells (5–7). In contrast, signal peptides without this motif guide proteins to the cell pole (8). Many YSIRK motif-containing proteins, including those with a C-terminal LPXTG motif, are anchored to the bacterial surface via sortase-mediated processing (9, 10). First identified by Rosenstein and Götz in staphylococcal lipases, the YSIRK/GxxS motif is also present in signal peptides of various *Streptococcus* and *Enterococcus* proteins but absent in other Firmicutes, such as *Bacillus* (11). One such protein with an extended signal peptide containing the YSIRK/GxxS motif is staphylococcal protein A (SpA), a multifunctional virulence factor of *S. aureus*. This motif ensures that SpA is specifically trafficked to the membrane of the dividing cross-wall, i.e., the septal membrane (5, 7). In a previous study, we demonstrated that this pre-targeting function of SpA correlates with the degradation of a SecA-associated peptidase, PepV (12). Here, we extend these findings by further examining the impact of SpA SP interaction with PepV.

PepV is a dipeptidase belonging to the M20 metalloprotease family (M20A subfamily). Members of this family catalyze diverse reactions (13) and despite limited sequence similarity share structural characteristics, typically adopting a two-domain architecture comprising a catalytic domain and a lid (or dimerization) domain (14). Crystal structures of M20A subfamily members, such as glutamate carboxypeptidases, reveal their propensity to form dimers, with interdomain interactions stabilizing the oligomeric state (13, 14). However, while the monomeric crystal structure of PepV has been resolved, its oligomerization states remain unexplored.

In this study, we investigate the role of the SpA SP in modulating the oligomerization states of PepV. Using native polyacrylamide gel electrophoresis (PAGE) and size-exclusion chromatography (SEC), we identified multiple PepV oligomeric states both *in vivo* and *in vitro*. The presence of the SpA SP, containing the YSIRK/GxxS motif, disrupted the formation of multimeric states. Interestingly, this inhibitory effect was not observed with the canonical SasF signal peptide, which lacks the YSIRK/GxxS motif. Both the SpA and SasF signal peptides inhibited PepV dipeptidase activity, with the SpA SP exerting a stronger inhibition. These observations reveal an unrecognized role for signal peptides as modulators of protein oligomerization. Specifically, our results suggest a broader functional role for signal peptides, particularly those containing the YSIRK/GxxS motif, in regulating protein activity and structural organization. This study highlights how signal peptides may extend beyond targeting functions to influence enzymatic activity and protein assembly, shedding new light on their multifaceted roles in bacterial physiology.

## RESULTS

### Modulation of PepV homomeric assembly by the SpA precursor and signal peptide

**Fig 1:**
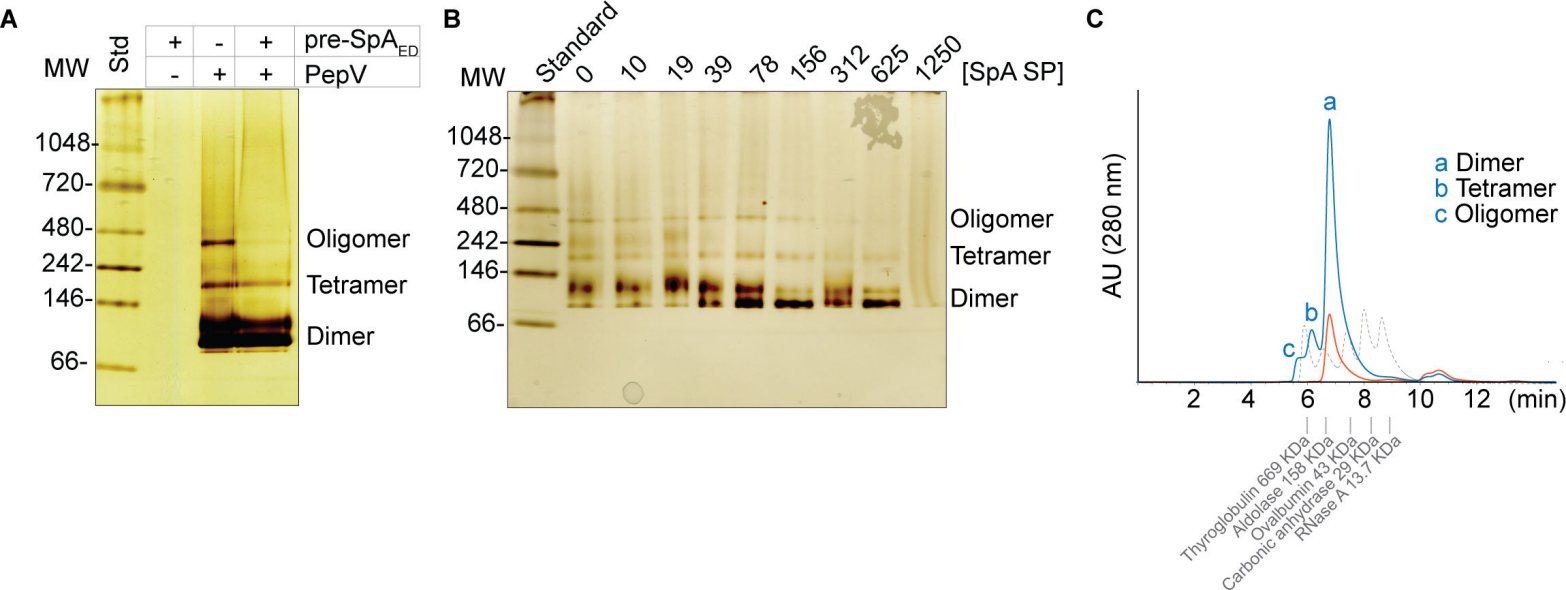
PepV oligomerization states and their modulation by the SpA precursor and signal peptide (SP). (**A**) Native PAGE analysis of purified PepV shows that it forms dimers, tetramers, and higher-order oligomers. In lane 3, PepV migrates without the SpA SP, while in lane 4, the presence of a 10-fold molar excess of SpA SP disrupts the tetrameric and higher-order species. Molecular weight markers on the left indicate the approximate sizes of these oligomeric forms. The *in vitro* translated SpA_ED_ precursor (~17 kDa), being a small peptide, is detectable only after extended silver stain development. (**B**) Dose-dependent effect of the SpA SP on PepV oligomerization. Purified PepV was incubated with increasing concentrations of the SpA SP and resolved on a gradient native gel. The progressive disappearance of tetrameric and oligomeric species with increasing signal peptide concentrations was visualized using silver staining. (**C**) Size-exclusion chromatography was performed to analyze PepV alone (blue) and PepV incubated with a 10-fold molar excess of SpA SP (red) in an aqueous buffered solution. Protein standards, along with their corresponding molecular weights, are shown in gray for reference.

In our previous study, we identified PepV as a modulator of SpA surface display (12). Building on this finding, we uncovered a negative regulatory loop in which the SpA precursor not only modulated PepV activity but also promoted its autodegradation (12). Given that PepV belongs to the M20 protease family, which typically forms homodimeric structures, we hypothesized that PepV might assemble into higher-order multimeric forms influenced by the SpA precursor (Table 1). To test this hypothesis, we analyzed purified PepV using native PAGE. The analysis revealed three distinct migrating species: a faster-migrating band of approximately 120 kDa (based on the native protein standard), an intermediate species corresponding to the tetrameric molecular weight of PepV, and the slowest-migrating band representing the oligomeric form, suggesting that the SpA precursor may regulate the oligomeric state of PepV (Fig. 1A).

**TABLE 1:** Amino acid sequences of peptides used in this study^*a*^

Peptides	Description	Sequence (N–C)
SpA SP	SpA SP	MKKKNIYSIRKLGVGIASVTLGTLLISGGVTPAANA
12 peptide	Portion of the SpA SP	YSIRKLGVGIAS
14 peptide	Portion of the SpA SP	IYSIRKLGVGIASV
24 peptide	Portion of the SpA SP	MKKKNIYSIRKLGVGIASVTLGTL
SasF SP	SasF signal peptide	MAKYRGKPFQLYVKLSCSTMMATSIILTNILPYDAQA
CWSS(−LPXTG)	SpA cell wall spanning region without the LPXTG motif	EENPFIGTTVFGGLSLALGAALLAGRRREL

^*a*^ CWSS, cell wall spanning sequence.

To examine whether the SpA precursor peptide affects PepV oligomerization, we incubated 0.5 µM PepV with a 10-fold molar excess of *in vitro*-translated SpA_ED_ precursor, a truncated form of SpA containing the signal peptide and the first two IgG-binding domains. Interestingly, under these conditions, the tetrameric and oligomeric forms of PepV disappeared (Fig. 1A). This observation raised the possibility that the signal peptide, rather than the IgG-binding domains, might be responsible for modulating the oligomeric states of PepV. To investigate this hypothesis, we incubated PepV with increasing concentrations of synthesized SpA SP and resolved the samples on a native gel. As shown in Fig. 1B, the tetrameric and oligomeric species gradually diminished at approximately 300 nM of SpA SP and became undetectable at higher concentrations, suggesting that the SpA SP can inhibit the dimeric and oligomeric states of PepV.

We next investigated whether SEC could be used to assess PepV multimerization and its inhibition by the SpA SP. Consistent with the results from native PAGE, SEC analysis of PepV revealed the presence of dimeric, tetrameric, and higher-order oligomeric species. When PepV was analyzed in the presence of the SpA SP, the chromatogram predominantly showed dimeric species of PepV (Fig. 1C). Note that the overall absorbance at 280 nm for PepV was lower in the PepV-SpA SP mixture compared to the PepV-alone solution. We speculate that this decrease in absorbance may result from a conformational change in PepV induced by its interaction with the SpA SP.

### Modulation of PepV multimeric forms by truncated SpA SPs

**Fig 2:**
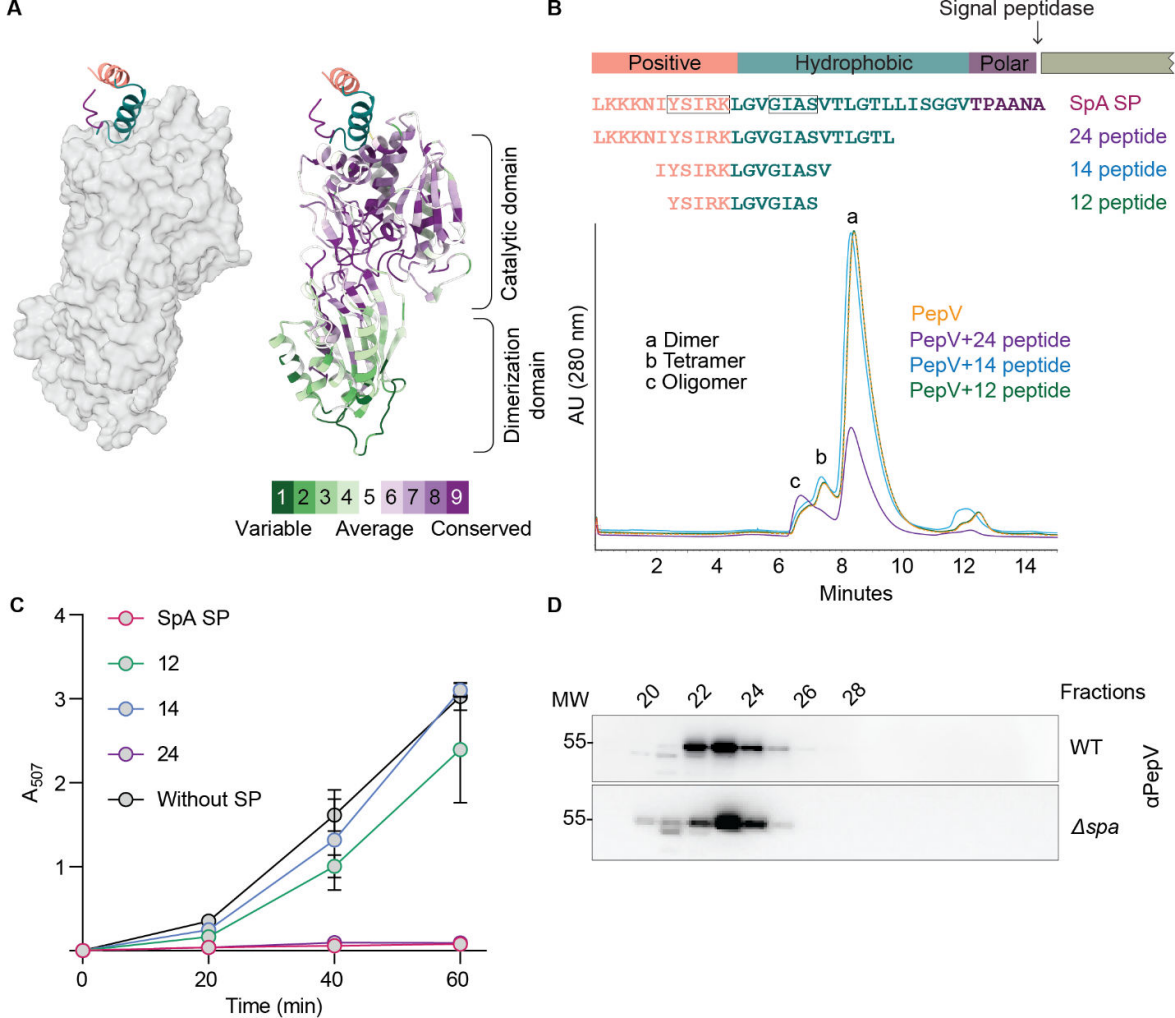
Truncated SpA SP variants show minimal impact on oligomerization and enzymatic activity. (**A**) Predicted structure of the SpA SP (SP) bound to PepV, showing potential interaction sites. The three SP motifs are color-coded to match the schematic in panel B. PepV is shaded according to amino acid conservation, based on phylogenetic analysis of homologs via the ConSurf server. (**B**) Schematic representation of truncated SpA SPs utilized in the SEC analysis, highlighting the regions covered by each variant and SEC absorbance chromatograms of PepV incubated with the truncated SpA SPs. The 24-residue peptide, encompassing the positively charged N-terminal motif and the majority of the hydrophobic motif, induced significant changes in the chromatogram, suggesting an interaction with PepV. Shorter peptides did not produce any observable effect on the PepV chromatogram. (**C**) Cd-ninhydrin assay of PepV enzyme incubated with 10 molar ratios of SpA SPs and its truncated variants. At the indicated time points, aliquots from each reaction (*n* = 2) were collected and immediately frozen in dry ice. After the final 60 min time point, ninhydrin reagent was added to all samples, and absorbance was measured at 507 nm to estimate PepV dipeptidase activity. Error bars denote standard deviations. (**D**) SEC elution profiles of soluble lysates from staphylococcal cells. The elution profiles of wild-type (WT) (top panels) and Δ*spa* (bottom panels) cells were analyzed. Eluted fractions were separated by SDS-PAGE, and PepV and SpA were detected via immunoblotting.

Given that the signal peptide of SpA influences the oligomeric steady state of PepV and that signal peptides are characterized by a tripartite motif structure—positive, hydrophobic, and polar—and considering that YSIRK/GXXS signal peptides also share these three motifs, we sought to further narrow down the sequence space and identify the specific motif(s) within the SpA SP that interact with PepV. To this end, we attempted to predict the structure of the SpA SP using AlphaFold (15). However, our efforts were not entirely successful, as AlphaFold was trained on protein structures that are well represented in the Protein Data Bank (PDB). Since signal peptides are often excluded from these structures, AlphaFold has not been adequately trained to predict them with high accuracy. To overcome this limitation, we used the CPH3 Model Server, a structure prediction tool that employs a remote-homology modeling algorithm when no suitable PDB template is identified, to predict the structure of the SpA SP (16). We then employed AlphaFold to model the complex structure of the SpA SP with a PepV monomer. AlphaFold predicted that the SpA SP interacts with the top of PepV’s catalytic domain, as illustrated in Fig. 2A. Within this interaction, the hydrophobic motif of the signal peptide (highlighted in teal in both Fig. 2A and B) is predominantly buried within a pocket on PepV. Interestingly, this pocket is composed of residues exhibiting neutral to weak evolutionary conservation, as determined using the ConSurf tool (17). To test the AlphaFold prediction, we incubated PepV with three truncated forms of the SpA SP: peptides 12, 14, and 24, composed of 12, 14, and 24 residues, respectively. These peptides partially represent the polar and hydrophobic motifs of the SpA SP. Among them, the 24-residue peptide includes the full N-terminal positive motif and approximately two-thirds of the hydrophobic motif (composed of 13 out of 19 amino acid residues of the hydrophobic motif). When PepV was incubated with a 10-fold molar excess of these peptides, distinct differences in the SEC profiles were observed (Fig. 2B). The SEC profiles for PepV incubated with the 12- and 14-residue peptides closely resembled that of PepV alone, suggesting minimal interaction. In contrast, the profile for PepV incubated with the 24-residue peptide exhibited some changes, with the tetrameric peak becoming indistinct.

To further probe the functional impact of these interactions, we assessed the enzymatic activity of PepV in the presence of the SpA SP and its truncated variants using Ala-Ala dipeptide as a substrate. Hydrolysis of the dipeptide was quantified via the Cd-ninhydrin colorimetric assay at 20, 40, and 60 min (Fig. 2C) (18). Consistent with the structural predictions, the peptidase activity was significantly affected by the full-length SpA SP and the 24-residue peptide, while peptides 12 and 14 exhibited negligible effects on enzymatic activity (Fig. 2C).

These findings collectively indicate that the SpA SP affects PepV multimerization. To determine whether this SpA precursor-mediated effect is also observed *in vivo*, we analyzed soluble cell lysates from WT and Δ*spa* strains using SEC. As shown in Fig. 2D, the absence of SpA resulted in a stronger PepV immunoblot signal at earlier high-molecular-weight fractions (fractions 20 and 21) compared to the WT lysate profile. This pattern aligns with the SEC profile of purified PepV and the SpA SP, further supporting a role for SpA precursor in modulating PepV multimerization.

### Roles of signal peptides and a non-signal peptide in PepV multimerization and dipeptidase activity

**Fig 3:**
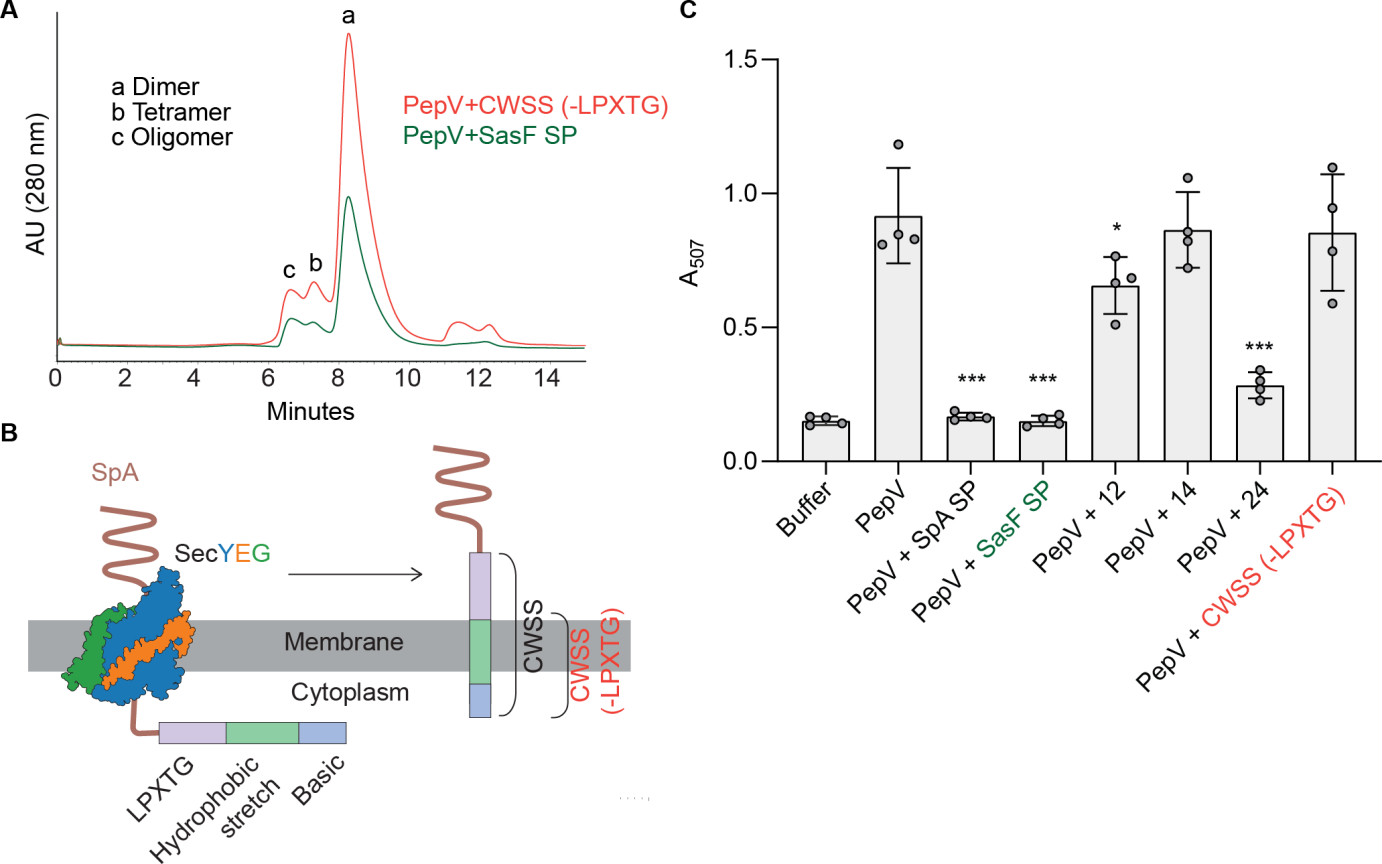
A non-YSIRK/GXXS signal peptide reduces PepV enzymatic activity without inhibiting oligomerization. (**A**) Size-exclusion chromatography (SEC) profile of PepV incubated with a 10-fold molar excess of the SasF signal peptide, a non-YSIRK/GXXS signal peptide, and the SEC absorbance profile of PepV incubated with the cell wall spanning sequence (CWSS) (-LPXTG) region of SpA. (**B**) A cartoon depicting the (-LPXTG) region of SpA, highlighting the hydrophobic domain and charged tail, excluding the LPXTG motif. (**C**) Enzymatic activity of PepV pre-incubated with signal peptides, truncated signal peptide variants, and the CWSS(−LPXTG) peptide of SpA. A two-tailed *t*-test was conducted to determine the *P* value, with significance levels denoted as follows: *P* values less than 0.05 are indicated by a single asterisk (*), while *P* values less than 0.001 are marked with three asterisks (***). Error bars denote standard deviations.

SpA SP, characterized by the YSIRK/GXXS motif, represses the higher-order oligomeric states of PepV and inhibits its dipeptidase activity (Fig. 1C and 2C). To further investigate the role of signal peptides in the modulation of multimeric states of PepV, we examined whether a signal peptide from a staphylococcal surface protein lacking the YSIRK/GXXS motif would have a similar effect on PepV oligomerization and enzymatic function. We selected the signal peptide of SasF, which does not contain the YSIRK/GXXS motif but is comparable in length to SpA SP; SasF consists of 37 residues, while SpA contains 36 residues. When PepV was incubated with the SasF signal peptide, the SEC profile revealed peaks corresponding to dimeric, tetrameric, and oligomeric forms of PepV (Fig. 3A), indicating that the SasF signal peptide does not repress PepV oligomerization.

Next, we asked whether the repression of PepV tetrameric and oligomeric forms could be achieved by a non-signal peptide entity containing both hydrophobic and basic amino acid motifs. We utilized the cell wall spanning sequence (CWSS) of SpA, which begins with the LPXTG anchoring motif and is followed by a membrane-spanning hydrophobic domain and a basic motif at the C-terminal end. Note that, while the CWSS peptide contains both hydrophobic and basic motifs, their arrangement differs from that of a signal peptide; the basic motif is positioned at the C-terminus of the hydrophobic motif. Upon incubating PepV with the CWSS(−LPXTG) peptide and analyzing its SEC profile, we observed peaks corresponding to dimeric, tetrameric, and oligomeric forms of PepV (Fig. 3A), suggesting that the CWSS(−LPXTG) peptide, despite containing hydrophobic and basic motifs, does not repress PepV oligomerization (Fig. 3B). While the SpA and SasF signal peptides had different effects on PepV oligomerization, both were able to inhibit its dipeptidase activity. In contrast, the CWSS peptide had no effect on enzymatic function (Fig. 3C).

Collectively, these findings suggest that repression of PepV tetrameric and higher-order oligomeric forms is specifically mediated by YSIRK/GXXS-type signal peptides. However, inhibition of dipeptidase activity can be induced by both YSIRK/GXXS-containing and canonical Sec signal peptides. Notably, the SEC profile (Fig. 3A) indicates that the non-YSIRK/GXXS signal peptide may alter the conformation of PepV, potentially reducing enzymatic activity without fully disrupting its oligomerization capacity. These observations highlight the need for future structural studies to delineate the conformational effects imparted by different signal peptides on PepV.

### PepV and its orthologs: multimeric states and dipeptidase activities

**Fig 4:**
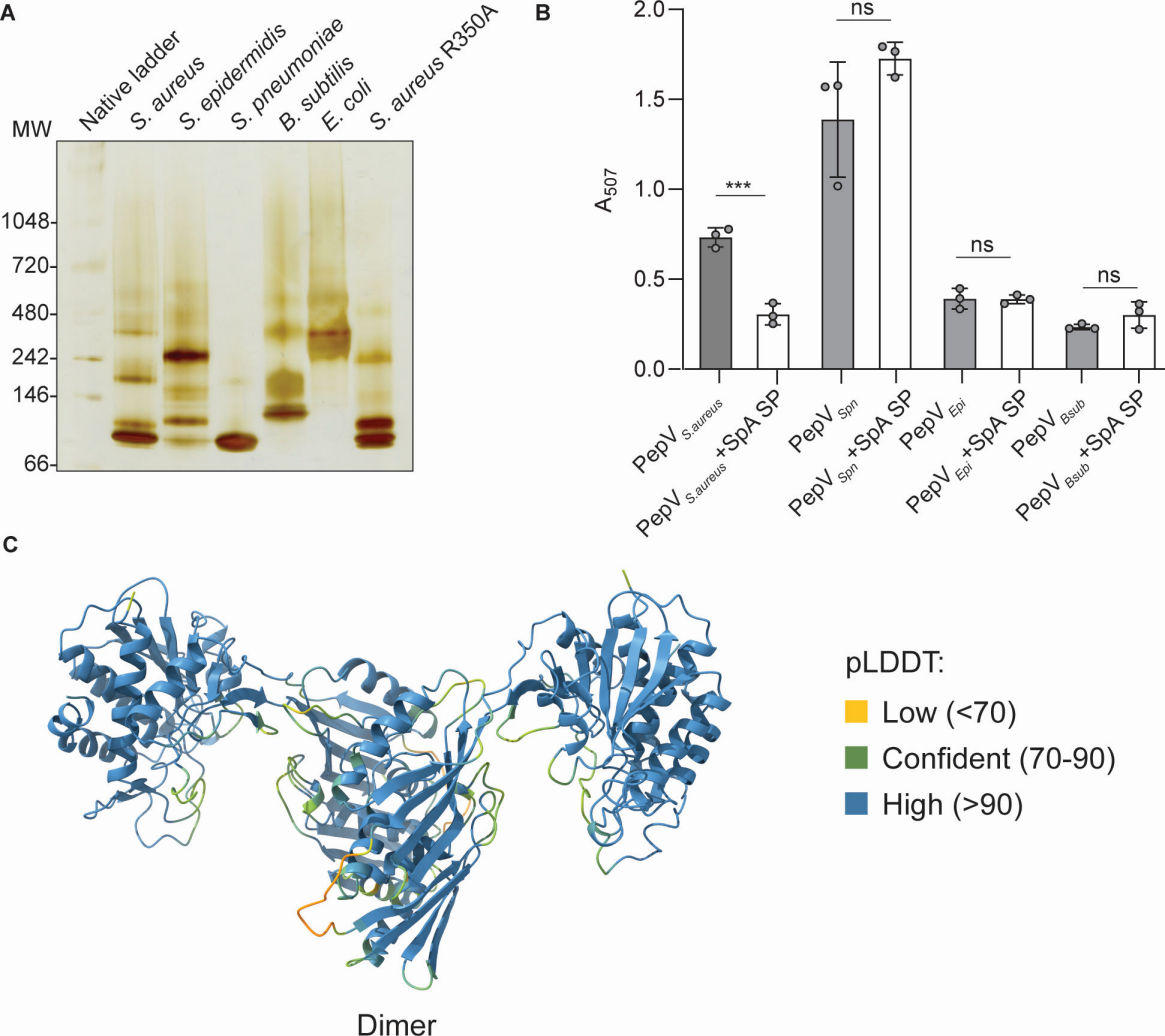
Multimerization potential and dipeptidase activities of PepV orthologs. (**A**) Multimeric states of purified PepV orthologs analyzed via native gel electrophoresis.(**B**) Inhibition of dipeptidase activity by the SpA signal peptide (SP) is observed exclusively with *S. aureus* PepV. Orthologs are denoted as follows: PepV*_Spn_* (*S. pneumoniae*), PepV*_Epi_* (*Staphylococcus epidermidis*), and PepV*_Bsub_* (*Bacillus subtilis*). Statistical significance was assessed using a two-tailed *t*-test. Error bars indicate standard deviation, and *** denotes *P* < 0.001. (**C**) AlphaFold-predicted structure of the PepV dimer with color coding based on calculated confidence scores (pLDDT) to indicate prediction reliability.

Staphylococcal PepV belongs to the peptidase subfamily M20A, as classified in the MEROPS database (19). Crystal structures of peptidases from subfamilies M20A and M20B reveal protein folds characteristic of clan MH, with homodimeric forms being predominant among the deposited structures. A notable exception is carboxypeptidase G2 from *Pseudomonas* sp., which exists both as a homodimer and as a tetramer (PDB ID: 1CG2) (20). Given these observations, we investigated whether PepV orthologs also exhibit multimeric assembly, such as dimers, tetramers, or higher-order oligomers. To identify PepV orthologs in representative bacterial species, we employed BLAST, using standard substitution matrices (BLOSUM 62 and BLOSUM 50). In cases where sequence similarity was absent, such as with *Esherichia coli* PepT, we used Foldseek to detect structurally similar proteins (21, 22). Our results indicate that, with the exception of the *Streptococcus pneumoniae* PepV ortholog, all identified orthologs formed multimeric assemblies beyond the dimeric state (Fig. 4A). Notably, *E. coli* PepT resolved as a tetramer in native gel analysis. The factors underlying these differences in multimerization potential among PepV orthologs remain unknown and warrant further investigation.

Given the AlphaFold prediction suggesting that the SpA SP interacts with PepV at a non-conserved pocket within the catalytic domain (Fig. 2A), we next examined whether SpA SP-mediated repression of enzymatic activity extends to PepV orthologs. To assess this question, we incubated a 10-fold molar excess of SpA SP with staphylococcal PepV and performed a colorimetric ninhydrin dipeptidase assay using Ala-Ala as the substrate (18). Our results showed that while SpA SP inhibited the dipeptidase activity of staphylococcal PepV, it had no effect on the enzymatic activities of PepV orthologs (Fig. 4B). These findings suggest that the inhibitory effect of SpA SP is specific to staphylococcal PepV and is not conserved among its orthologs.

The monomeric crystal structure of PepV has been resolved and is available in the Protein Data Bank (PDB ID: 3KI9). However, predicting its multimeric structures using AlphaFold yielded mixed results (Fig. 4C). The monomeric structure, within the predicted dimer, was predicted with high confidence, with per-residue average local accuracy scores (pLDDT) exceeding 90 and a moderate global confidence score (pTM ~0.6) (23). In contrast, the overall dimeric structure was predicted with moderate confidence (Fig. 4C), as the confidence in the dimeric interface was relatively low (iPTM ~0.3). A potential reason for this challenge lies in the distinctive dimerization domain of PepV. In homologous M20 proteases, dimerization typically occurs through a conserved interface comprising four β-strands and two α-helices, a structural hallmark of the MEROPS peptidase family M20 (24). In contrast, PepV features a duplicated dimerization domain, incorporating eight β-strands and four α-helices. This structural divergence may contribute to the difficulty of AlphaFold in accurately predicting the dimeric interface with high confidence (Fig. 4C). AlphaFold’s tetrameric prediction was also of low confidence (structure not shown). Of the five models generated, one resembled the resolved tetramer of the M20 peptidase carboxypeptidase G2 (PDB ID: 1CG2), suggesting a possible tetrameric arrangement for PepV. However, the low confidence highlights the need for experimental validation of its multimeric architecture.

## DISCUSSION

Our study provides evidence that staphylococcal PepV, a dipeptidase, assembles into multimeric structures, including dimers, tetramers, and higher-order oligomers. Notably, we find that SpA SP selectively inhibits PepV enzymatic activity while also suppressing its tetrameric and oligomeric states. While SpA SP influences both multimerization and enzymatic function, the precise relationship between these structural states and their impact on PepV activity remains unresolved. Furthermore, we demonstrate that PepV orthologs similarly form multimeric assemblies, suggesting that multimerization is a conserved feature across species. Collectively, our findings indicate that signal peptides may serve functions beyond their canonical role in protein translocation, potentially acting as regulators of protein structure and activity.

To the best of our knowledge, non-targeting functions of signal peptides have been previously reported; however, these functions are typically linked to protein translocation. For example, signal sequences can influence interactions with the translocon or regulate membrane insertion efficiencies, thereby affecting the efficiency of protein targeting (2, 25). Beyond their role in translocation, signal peptides have also been implicated in other cellular processes. One notable example is the signal sequence of preprolactin, which undergoes cleavage upon insertion into the endoplasmic reticulum membrane. The resulting N-terminal fragment is subsequently released into the cytosol, where it interacts with Ca²^+^ and calmodulin, suggesting a role in intracellular signaling (26, 27). Signal peptides also play an immunological role through presentation by major histocompatibility complex class I molecules. Human HLA-A, HLA-B, and HLA-C bind peptides from pathogens or cellular proteins, including those derived from signal sequences, for recognition by cytotoxic T cells, underscoring their role beyond protein targeting (28). A striking example from a Gram-positive bacterium is the pheromone peptide produced from the signal sequence region of the lipoprotein CcfA in *Enterococcus* (29). Despite these insights, the involvement of signal peptides in directly modulating or regulating enzyme activity has not been reported in bacterial systems. Our findings, in conjunction with previous studies, suggest that the functional scope of signal peptides extends beyond mere targeting, potentially encompassing regulatory roles in cellular processes.

Several unresolved questions emerge from this study. First, why does the SpA SP alter PepV multimerization (Fig. 1C), whereas the SasF SP does not (Fig. 3A)? Second, can the variation in multimerization observed among PepV orthologs (Fig. 4A) be predicted from sequence differences in their dimerization domains? At present, our data do not provide definitive answers, and detailed structural studies will be required to address these issues. However, despite the high sequence similarity of the dimerization domains in *S. aureus*, *Staphylococcus epidermidis*, and *S. pneumoniae* PepV (Fig. S1), their multimerization potential differs (Fig. 4A). This observation suggests that features outside the dimerization domain may also contribute to the regulation of multimerization.

The biological significance of the regulatory activity of SpA SP remains an intriguing question. In a previous study (12), we observed that the absence of PepV leads to increased SpA localization at the septum, suggesting enhanced surface display of SpA. In the same study, we found that cells lacking the *pepV* gene were slightly smaller than the wild-type (WT) cells. Taken together, these observations suggest the presence of a negative regulatory loop that coordinates both cell size and surface protein display. Within this loop, PepV negatively regulates SpA surface display, while the SpA SP, in turn, inhibits the enzymatic activity and multimerization potential of PepV, thereby counteracting its suppressive effects. This dynamic interplay between SpA and PepV appears to function as a molecular “tug-of-war,” possibly fine-tuning growth and ensuring the appropriate levels of SpA anchoring at the septum.

## MATERIALS AND METHODS

### Bacterial culture, strains, and plasmids

Strains and plasmids used in this study are listed in Table 2, and primers are listed in Table 3. *S. aureus* strain RN4220 and derivatives were used for all experiments (30). *S. aureus* was cultured in tryptic soy broth or on tryptic soy agar plates. *Escherichia coli* BL21(DE3) was used for production of histidine-tagged proteins. To obtain soluble lysates for high-performance liquid chromatography (HPLC), bacterial cultures were grown to mid-exponential phase before cells were harvested by centrifugation and resuspended in buffer A [0.5 M 4-(2-hydroxyethyl)-1-piperazineethanesulfonic acid (HEPES), 150 mM NaCl, and EDTA-free protease inhibitor; Sigma]. Cell lysis was achieved via bead beating, followed by centrifugation to remove cell debris. The soluble fraction was further clarified by ultracentrifugation at 100,000 × *g* for 1 h.

**TABLE 2:** List of strains and plasmids used in this study

	Genotype/description	Source/references
Plasmid (name/designation)		
pSY273	pET16b-*H6-pepV*	(12)
Strains (name/designation)		
Wild type	*S. aureus* RN4220 *sau1 hsdR*, a derivative of NCTC8325-4	(30, 31)
Δ*pepV* (SA2101)	RN4220 Δ*pepV*	(12)

**TABLE 3:** Oligonucleotides used in this study

Oligo	Description	Sequence 5′−3′
OSA1088	T7 promoter plus the transcription start site of *spa*	TAATACGACTCACTATAGGGACGTAGGAGATATAC CATGAAAAAGAAAAACATTTATTCAATTCGTAAACTAGGTGTAGG
OSA1089	Reverse primer, base pairs at the end of IgG-binding domain D	TTATTTCGGTGCTTGAGATTCGTTTAATTTTTTAGC

### Protein purification

Recombinant His-PepV proteins were produced using *E. coli* BL21(DE3). Following IPTG induction, bacterial cells were harvested (10,000 × *g*, 10 min), washed, and resuspended in buffer A (50 mM Tris-HCl, pH 7.5, 150 mM NaCl, and 10 mM imidazole). Cells were lysed using two passages through a French press at 14,000 lb/in². Unbroken cells were removed by centrifugation (5,000 × *g*, 15 min), and the resulting crude lysates were further clarified by ultracentrifugation (100,000 × *g*, 1 h, 4°C). The soluble fraction was applied to a Ni-NTA resin column (Qiagen) pre-equilibrated with buffer A. The column was washed with 20-bed volumes of buffer A, and proteins were eluted in 2 mL fractions using a step gradient of imidazole (20–500 mM). Aliquots from different purification steps, including column loading, flow-through, washes, and eluted fractions, were mixed with sample buffer and analyzed by SDS-PAGE (12% or 15%). Proteins were visualized by Coomassie Brilliant Blue staining or Western blotting. Fractions containing the protein of interest were pooled, dialyzed in phosphate-buffered saline, and stored at −80°C until further use. For *in vitro* translation, template DNA was generated by PCR using the OSA1088/OSA1089 primer pair, with the T7 promoter sequence included at the 5′ end of the forward primer (OSA1088). Translation was carried out using the NEB PURExpress kit according to the manufacturer’s instructions. The translated SpA_ED_ precursor was purified using IgG Fast Flow affinity resin (Cytiva) following the manufacturer’s protocol.

### Size-exclusion chromatography and immunoblotting

Size-exclusion chromatography was performed using Agilent SEC-3 (Fig. 1) and TSKgel Super2000 (Fig. 2 and 3) columns mounted on a Waters e2695 HPLC system. Before sample loading, the column was equilibrated with buffer (0.5 M HEPES, pH 7.6, and 150 mM NaCl). The protein sample was passed through the column at a flow rate of 0.35 mL/min. Protein standards (CellMosaic) were analyzed under identical conditions, and absorbance was monitored at 280 nm. Collected SEC fractions were mixed with SDS sample buffer and subjected to SDS-PAGE for protein resolution. Following electrophoresis, proteins were electro-transferred onto a nitrocellulose membrane. The membranes were then blocked for 30 min with a 10 mL blocking solution containing 5% milk and, when required, 70 µL of human IgG (Sigma). After blocking, membranes were incubated with primary antibodies for 1 h followed by three 10 min washes with TBST (50 mM Tris-HCl, pH 7.5, 150 mM NaCl, 0.1% Tween 20) to remove unbound antibodies. Next, membranes were treated with a horseradish peroxidase-conjugated secondary antibody and washed three times with TBST. Protein detection was carried out using SuperSignal Chemiluminescent Substrate (Thermo Fisher Scientific).

### Native gel electrophoresis

The protein mixtures were prepared by adding native sample buffer (62.5 mM Tris, pH 6.8, 20% glycerol, and 0.05% bromophenol blue) before being loaded onto a NativePAGE 4%–16% gel (Thermo Fisher Scientific). Electrophoresis was carried out at a constant current of 5 mA at 4°C to ensure optimal separation under native conditions. Following electrophoresis, the gels were stained using Pierce Silver Stain for Mass Spectrometry (Thermo Fisher Scientific), according to the manufacturer’s instructions.

### Cd-ninhydrin assay

The dipeptidase activity of PepV was assessed using the Cd-ninhydrin method, following a previously established protocol (18). To initiate the reaction, the Ala-Ala dipeptide substrate was incubated with PepV, along with the signal peptide and its variants, at 37°C for 30 min in a reaction buffer containing 25 mM Tris (pH 7.5), 2 mM MnCl_2_, and 5 mM tris(2-carboxyethyl)phosphine. The reaction was then terminated by the addition of Cd-ninhydrin reagent, followed by heating at 75°C for 10 min. Enzymatic activity was quantified by measuring absorbance at 507 nm.

## SUPPLEMENTAL MATERIAL

Fig. S1 spectrum.01778-25-s0001.docx Global alignment of PepV with representative orthologs.

ASM does not own the copyrights to Supplemental Material that may be linked to, or accessed through, an article. The authors have granted ASM a non-exclusive, world-wide license to publish the Supplemental Material files. Please contact the corresponding author directly for reuse.
