# Supplementary material for: The signal peptide of staphylococcal protein A alters the multimeric states of PepV
Source: Microbiol Spectr. 2025 Oct 27;13(12):e01778-25. doi: 10.1128/spectrum.01778-25 (PMC12671168; doi:10.1128/spectrum.01778-25)
Supplement: Fig. S1 — Global alignment of PepV with representative orthologs. [file spectrum.01778-25-s0001.docx]

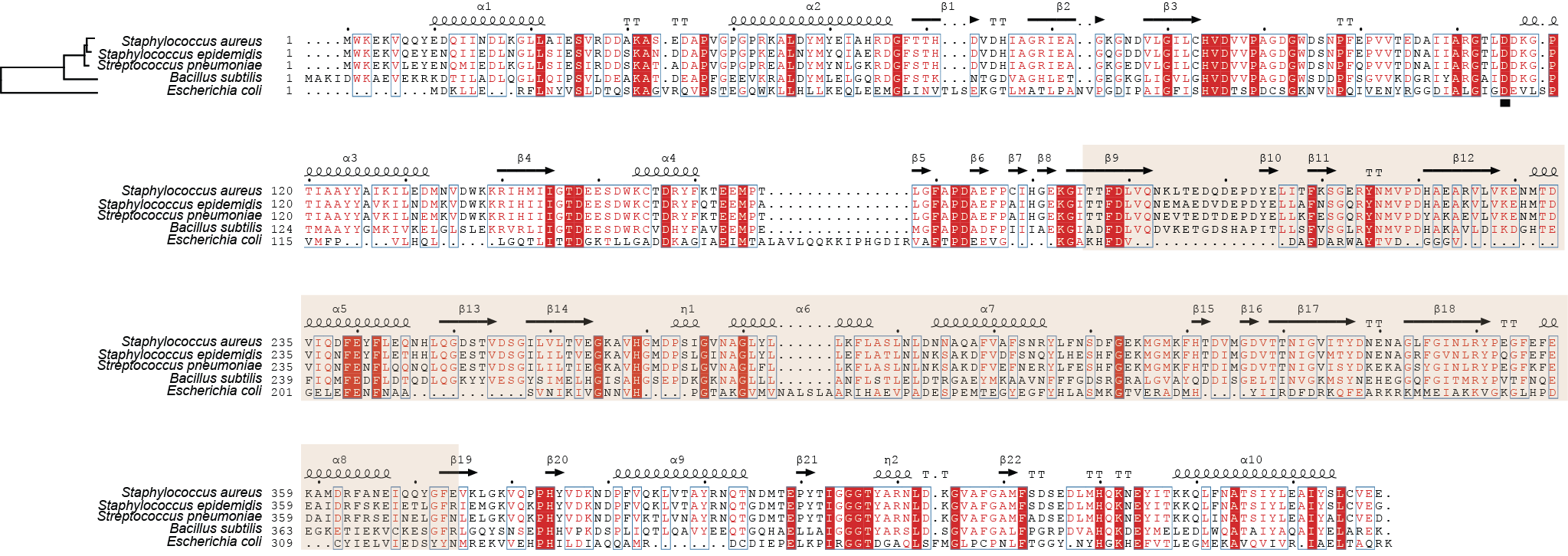


**FIG S1: Global alignment of PepV with representative orthologs.** The alignment was generated using the Clustal Omega algorithm (1) and visualized with the ESPript 3 web tool (2). α-helices are indicated by squiggles, β-strands by arrows, and strict β-turns by the letters TT. Regions corresponding to the dimerization domain are shaded in the alignment.

**REFERENCES**

1. Sievers F, Wilm A, Dineen D, Gibson TJ, Karplus K, Li W, Lopez R, McWilliam H, Remmert M, Söding J, Thompson JD, Higgins DG. 2011. Fast, scalable generation of high‐quality protein multiple sequence alignments using Clustal Omega. Molecular Systems Biology 7:539.

2. Robert X, Gouet P. 2014. Deciphering key features in protein structures with the new ENDscript server. Nucleic Acids Research 42:W320-W324.
